# KLHL3-dependent WNK4 degradation affected by potassium through the neddylation and autophagy pathway

**DOI:** 10.1186/s12882-023-03257-4

**Published:** 2023-07-22

**Authors:** Siqi Ying, Qin Guo, Chong Zhang

**Affiliations:** 1 Department of Nephrology, Jing’an District Center Hospital of Shanghai, Fudan University, Shanghai, 200040 China; 2 Department of Nephrology, Xin Hua Hospital Affiliated to Shanghai Jiao Tong University School of Medicine, Shanghai Shi, China

**Keywords:** KLHL3, CUL3, WNK4, Potassium, Neddylation, Autophagy

## Abstract

**Background:**

Studies reported that kelch-like protein 3 (KLHL3)-Cullin3(CUL3) E3 ligase ubiquitinated with-no-lysine kinase 4 (WNK4). Impaired WNK4 ubiquitination plays a key role in Familial hyperkalemic hypertension (FHHt, also called pseudohypoaldosteronism type II) which results from overaction of thiazide-sensitive sodium chloride cotransport (NCC). In addition, researchers have also found that dietary potassium deficiency activates NCC along the renal distal convoluted tubule (DCT). However, the underlying mechanism remains unclear about the relationship between potassium and WNK4.

**Methods:**

In the present study, we conducted in vitro and in vivo experiments to confirm that KLHL3-dependent WNK4 degradation is affected by potassium through the neddylation and autophagy pathway. In vitro, the WNK4 and KLHL3 plasmids were cotransfected into HEK293 cell lines by lipofectamine 2000, and then incubated with different potassium concentrations (1mmol/L and 10mmol/L) for 24 h, and further treated with MLN4924 or the autophagy inhibitor or both of MLN4924 and the autophagy inhibitor for another 24 h respectively. In vivo, we created mice that were fed with low or high potassium diets and then were injected MLN4924 in the experimental groups. The expression of WNK4, pWNK4, KLHL3, NEDD8, LC3 ,and P62 was detected by western blotting in vitro and vivo experiments.

**Results:**

We found that the abundance and phosphorylation of WNK4 increase when neddylation is inhibited both in vitro and vivo. Furthermore, the abundance of pWNK4, WNK4, NEDD8, and KLHL3 was increased in the low potassium (LK) group. Inhibiting autophagy can ameliorate the effect of potassium on the abundance and activity of WNK4 to some extent.

**Conclusion:**

These findings suggest a complex regulation of potassium in the degradation of WNK4. Low potassium can activate WNK4, which may be related to neddylation and autophagy, but the mechanism needs to be further studied.

**Supplementary Information:**

The online version contains supplementary material available at 10.1186/s12882-023-03257-4.

## Introduction

Pseudohypoaldosteronism type II (PHAII), also known as “familial hyperkalemic hypertension (FHHt)” or “Gordon’s syndrome” is a rare Mendelian syndrome characterized by hypertension, hyperkalemia, metabolic acidosis ,and hypercalciuria, which results from the overactivation of thiazide-sensitive sodium chloride cotransport(NCC) [1, 2]. NCC can be directly phosphorylated by Ste20related proline alaninerich kinase (SPAK) and oxidative stress response 1 kinase (OSR1) at conserved threonine and serine residues in the aminoterminal domain, while with-no-lysine kinase1(WNK1) and WNK4 activate SPAK/OSR1 [3, 4]. Up to date, four mutated genes have been implicated in FHHt: two coding for kinases (WNK1 and WNK4) and the other two coding for cullin-RING ligase(CRL) proteins kelchlike 3 (KLHL3) and cullin3 (CUL3). The products of these genes all appear to modulate NCC [5]. CUL3, a scaffold protein, connects the substrate adaptor protein and RING-box1 ubiquitin ligase E3 in the cullin-RING ubiquitin E3 ligase complexes [6]. KLHL3, a member of the BTB-BACK-Kelch family, interacts with CUL3 to form the E3 ubiquitin ligase complex [7]. KLHL3 combines with WNK kinases, targeting them for ubiquitylation and proteasomal degartion [8–10]. WNK4 is a target for ubiquitination by the KLHL3-CUL3 E3 ligase complex [10, 11]. CRL must be activated before ubiquitylation occurs via neddylation in which a NEDD8 (neuronal precursor cell expressed developmentally downregulated protein 8) is covalently attached to the cullin [12–14].

Autophagy ubiquitously occurs in eukaryotic cells, which is a biological process of self-repair that concerns cellular growth, metabolism, and defenses against oxidative stress [15]. Kelch-like ECH-associated protein1(Keap1), which belongs to the same kelch family as KLHL3, is an important component of an E3 ligase complex that ubiquitinates the transcription factor, nuclear factor erythroid 2-related factor 2 (Nrf2) [16]. P62/SQSTM1 is a ubiquitin-binding autophagy receptor protein and links the Nrf2 pathway and autophagy [15]. There is a study showing that selective autophagy is mediated by p62/SQSTEM1 in KLHL3-dependent WNK4 degradation [16]. In the present study, we have investigated that neddylation and autophagy participate in KLHL3-dependent WNK4 degradation in vitro and vivo.

## Materials and methods

### Plasmids and cell lines

KLHL3, CUL3, and WNK4 plasmids were donated by David Ellison, Division of Nephrology & Hypertension, Department of Medicine, Oregon Health & Science University. HEK293 cell lines (Preserved in the Department of Nephrology, Xinhua Hospital, School of Medicine, Shanghai Jiao Tong University, Shanghai, China).

### Animals

All animal studies were approved by the Shanghai Xinhua Hospital Ethics Committee and all methods were carried out under relevant guidelines and regulations. This study was carried out in compliance with the ARRIVE guidelines. All mice were 12–24 weeks old, 25-30 g, male ,and had a C57Bl/6 background. All strains are backcrossed to the appropriate wild-type mice every ten generations to maintain genetic backgrounds. The mice were divided into low-potassium (LK) and high-potassium (HK) feeding groups. After feeding for 3 months, the experimental group was injected with MLN4924 (30 mg/kg, s.c.), a protein neddylation inhibitor, dissolved in 10% 2-hydroxypropyl-β-cyclodextrin (HPBCD) twice a day (with an interval of 12 h) for three consecutive days [17]. The vehicle control group received an equivalent volume of 10% HPBCD (vehicle) in the same manner and at the same time as the MLN4924 group [18].

### Animal diets

All diets used in K^+^ studies were purchased from Rendy. Dietary manipulations included a low (0.005%) or a high (5%) K^+^ diet for the indicated period. NaCl content (0.3%) in a low-K^+^ diet is the same as that in a high-K^+^ diet [19, 20].

### Antibodies and reagents

MLN4924 (catalog no.A-1139; Activebiochem), 3-methyladenine (Sigma-Aldrich), Bafilomycin A1 (MedChemExpress), Lipofectamine 2000 (Invitrogen), protease inhibitor cocktail (CompleteMini, Roche), phosphatase inhibitor cocktail (PhosStop, Roche).

Immunoblots were probed using the following primary antibodies: anti cullin3 antibody (Cell Signaling Technology), anti-β-actin monoclonal antibody (Santa Cruz), anti-NEDD8 antibody (Cell Signaling Technology), anti-c-Myc antibody (Sigma-Aldrich), anti-p62 antibody (MBL), anti-light chain 3B antibody (LC3B; MBL and Cell Signaling Technology), anti-glyceraldehyde-3-phosphate dehydrogenase antibody (GAPDH; Cell Signaling Technology), anti WNK4 and anti pWNK4 antibody (from Oregon Health & Science University), anti Keap1 antibody (Santa Cruz), anti Nrf2 antibody (Santa Cruz). Alkaline phosphatase-conjugated anti-immunoglobulin G (IgG) antibodies (Santa Cruz) were used as the secondary antibodies for immunoblotting.

### Cell culture and transfection

HEK293 cells were cultured at 37℃ in a humidified 5% CO_2_ atmosphere in Dulbecco’s modified Eagle’s medium supplemented with 10% (v/v) FBS. HEK293 cells (3 × 10^5^cells/6-cm dish or 1 × 10^5^cells/3.5-cm) were transfected with the indicated amount of plasmids using the Lipofectamine 2000 reagent. For autophagy inhibition, cells were incubated with 3-methyladenine (3-MA) /Bafilomycin A1 for 24 h before harvesting.

### Western blotting

Transfected HEK293 cells were lysed in a buffer (50mM Tris/HCl, pH7.5, 150mM NaCl, 1%Nonidet P-40, 1mM sodium orthovanadate, 50mM sodium fluoride, protease inhibitor cocktail and phosphatase inhibitor cocktail ) for 30 min at 4℃. As for in vivo experiments, kidneys were harvested and snap-frozen in liquid nitrogen and then homogenized on ice in a chilled buffer containing protease and phosphatase inhibitors. Protein samples were separated by SDS-PAGE and transferred to polyvinylidene fluoride (PVDF) membranes (Roche) using the Trans-Blot Turbo transfer system (Bio-Rad Laboratories). Membranes were blocked with 5% nonfat milk in TBS-Tween for 1 h at room temperature, before incubation with the primary antibody in blocking buffers overnight at 4 °C.

### Statistical analysis

Statistical analysis of the research data was performed by using Graphpad 8.0 software. Comparisons between the two groups were performed using unpaired Student’s t-tests. *P* < 0.05 was considered statistically significant. Data are presented as the means ± S.E.M.s.

## Results and discussions

### Neddylation and autophagy are involved in the degradation of CUL3-KLHL3-WNK4

**Fig. 1:**
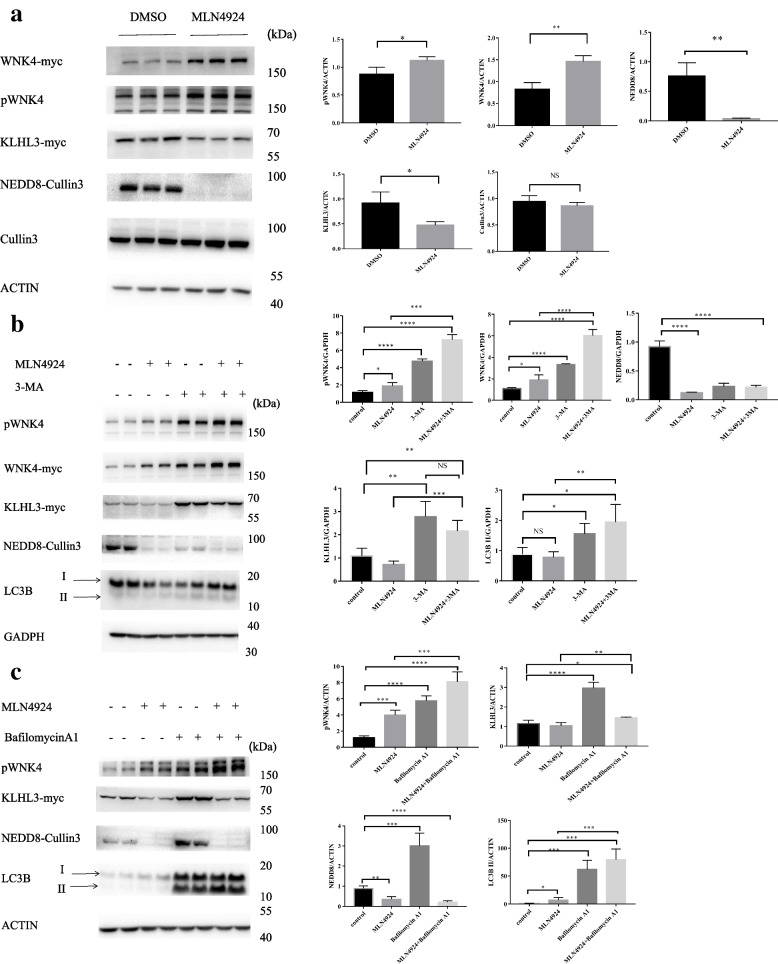
The neddylation and autophagy in the KLHL3-dependent WNK4 degradation. **a** Immunoblotting was performed with antibodies against WNK4, pWNK4, KLHL3-myc, NEDD8 ,and cullin3. Compared with control groups, the expression of WNK and pWNK4 increases significantly in the experimental groups. Densitometry analysis of WNK4, pWNK4, KLHL3, Nedd8 and Cullin3 . **P < 0.01, *P < 0.05 *n*= 6 **b** HEK293T cells were treated with an autophagy inhibitor (3MA) and MLN4924 separately and in combination for 24 h. The combined effect of 3MA and MLN4924 for WNK is stronger than the single effect. Densitometry analysis of WNK4, pWNK4, KLHL3, NEDD8 ,and LC3B.****P <0.0001, ***P < 0.001, **P < 0.01, *P < 0.05 *n*= 4 **c** Another autophagy inhibitor, Bafilomycin A1, is similar to 3MA

We examined whether neddylation and autophagy are involved in the KLHL3-dependent WNK4 degradation pathway. To test this, HEK293 cells co-expressing KLHL3 and WNK4 were treated with DMSO or MLN4924, respectively. We can find that the abundance and phosphorylation of WNK4 increase in the MLN4924 group (Fig. 1a). Interestingly, the abundance of KLHL3 decreased, which did not increase as expected (Fig. 1a). There is a study showing that the increase in CUL3 neddylation could promote the degradation of KLHL3 [21]. However, our results are contrary to the expected results, indicating that there exist other degradation pathways for KLHL3 in addition to neddylation. Unfortunately, phosphorylation of KLHL3 is not detected. It is known that phosphorylation of KLHL3 by PKA inhibited WNK4 degradation [22]. Therefore, we hypothesized that phosphorylated KLHL3 would be increased in the MLN4924 group which needs further experiments to verify. Besides, reports are showing that MLN4924 activates autophagy [23, 24].

To explore another degradation pathway, we performed the next experiment. We intervened in HEK293 cell lines co-expressing KLHL3 and WNK4 with an autophagy inhibitor (3-methyladenine, 3-MA or Bafilomycin A1, BA1) and MLN4924 separately and in combination. As a result, the abundance of pWNK4 and WNK4 increased when using two reagents compared to using one regent (Fig. 1b). As for KLHL3, our findings were the same as before. Furthermore, KLHL3 increased when MLN4924 and 3-MA were used in combination compared to using MLN4924 alone. What is more, the LC3B-II band was indeed increased with MLN4924 treatment as shown in Fig. 1b and c. Hence, we speculated that autophagy may regulate the degradation of WNK4 and KLHL3. Inhibiting autophagy can reverse the reduction of KLHL3 abundance caused by MLN4924. However, there is confusion that why the abundance of KLHL3 did not increase more when using MLN4924 and 3-MA in combination compared to using 3-MA alone (Fig. 1b). And the abundance of KLHL3 seems to decline, but it is not statistically significant (Fig. 1b). Regrettably, we did not detect the phosphorylation level of KLHL3, which should be explored in further experiments. When using a different autophagy inhibitor (Bafilomycin A1, BA1), it has a similar performance as 3-MA (Fig. 1c ).

### The effect of potassium on the CUL3-KLHL3-WNK4 cascade in vitro

Recent studies have demonstrated that extracellular potassium regulates the activity of NCC [25, 26]. Terker et al have convincingly proved that extracellular K^+^ regulates the activity of the WNK-SPAK signaling pathway in the DCT and that hyperkalemia inhibits, while hypokalemia stimulates NCC phosphorylation [27, 28]. Furthermore, an inwardly rectifying potassium channel 4.1 (Kir4.1) plays an essential role in mediating the effect of extracellular potassium intake on NCC activity and potassium homeostasis [26, 29]. WNK kinases are reported to be chloride-sensitive, and the intracellular chloride concentration [Cl^−^]_i_inhibits autophosphorylation and activity of WNK1 and WNK4 [27, 30, 31]. When the Kir4.1 channel is activated, intracellular K^+^ flows out of the cell from kir4.1, which hyperpolarizes DCT cells with a consequent chloride efflux. Therefore, [Cl^-^]_i_ is reduced and NCC is activated via a WNK4-SPAK-dependent mechanism.

**Fig. 2:**
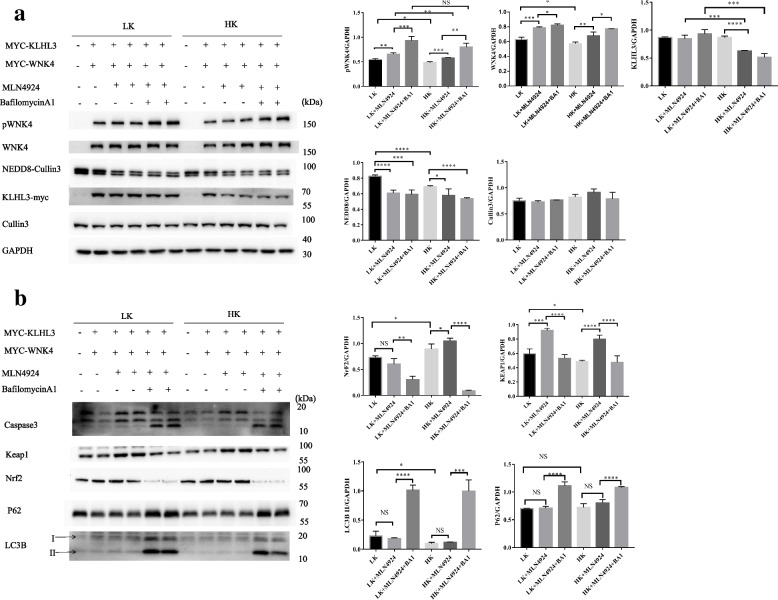
Potassium regulates WNK4 via autophagy and apoptosis. HEK293 cells co-expressed with KLHL3 and WNK4 kinase were treated by different potassium ion concentrations shown in **a** and **b**. It shows that the abundance of WNK4，pWNK4，NEDD8-Cullin3,capase3 ,and LC3B II increases in LK groups more than in HK groups. The expression of WNK4 and pWNK4 is higher with two drugs (MLN4924 and Bafilomycin A1) than MLN4924 only. Densitometry analysis of WNK4, pWNK4, KLHL3, NEDD8, CUL3, P62, KEAP1 and LC3B. ****P <0.0001, ***P < 0.001, **P < 0.01, *P < 0.05 *n*= 4

To verify the effect of potassium on the CUL3-KLHL3-WNK4 cascade, we performed the following experiments. We incubated HEK293 cells co-transfected with KLHL3 and WNK4 plasmids at different potassium concentrations (1mmol/L and 10mmol/L) for 48 hours. The results revealed that the abundance of pWNK4, WNK4, NEDD8, and KLHL3 was increased in the low potassium (LK,1mmol/L) group compared to the high potassium(HK,10mmol/L) group (Fig. 2a). Furthermore, MLN4924 increased the expression of WNK4, pWNK4, and Keap1 in the LK and HK groups. The abundance of WNK4 and pWNK4 was higher in the group using MLN4924 and Bafilomycin A1 in combination than MLN4924 alone, which might indicate that WNK4 can be degraded by autophagy. And inhibiting autophagy can ameliorate the effect of potassium on WNK4 to some extent, which suggests potassium regulates WNK4 through\ autophagy. However, the abundance of KLHL3 did not show an obvious increase when treated with MLN4924 and Bafilomycin A1 in combination both in the LK and HK group and decreased in HK groups compared to LK groups (Fig. 2a), from which we could assume that potassium might play the role of degradation of KLHL3 and high potassium seemed to counteract the autophagy-inhibiting effect of Bafilomycin A1. In conclusion, apart from neddylation and autophagy, there is another degradation pathway for KLHL3 affected by potassium.

Moreover, treatment groups showed significantly enhanced apoptosis and autophagy compared to the control groups both in the LK groups and HK groups (as shown in Fig. 2b). Unfortunately, we did not do further experiments on autophagy and apoptosis in this study.

### The effect of potassium on the CUL3-KLHL3-WNK4 cascade in vivo

**Fig. 3:**
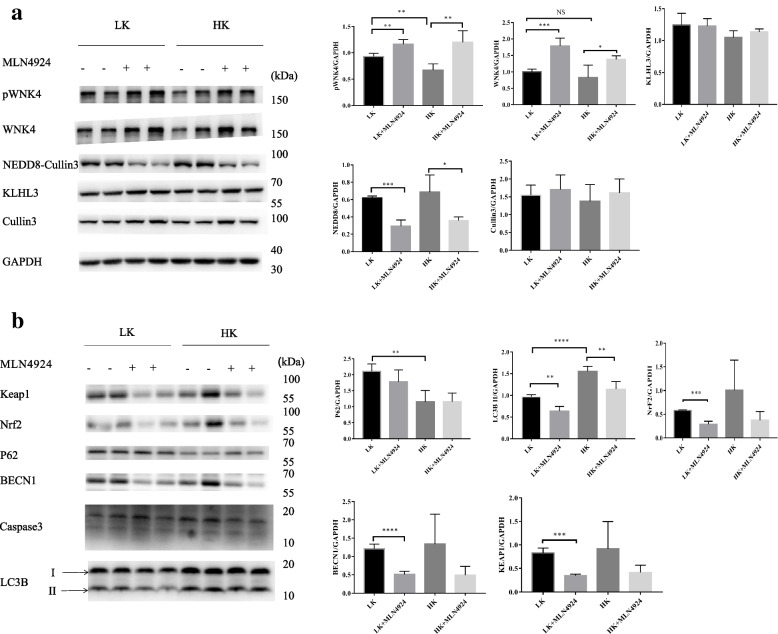
The effect of potassium on the CUL3-KLHL3-WNK4 cascade in vivo. Mice were fed with HK and LK diet and then were injected with MLN4924. Within LK groups or HK groups, the expression of pWNK4 and WNK4 increased with MLN4924 treatment. Densitometry analysis of WNK4, pWNK4, KLHL3, NEDD8, CUL3, P62, BECN1, KEAP1 and LC3B.****P <0.0001, ***P < 0.001, **P < 0.01, *P < 0.05 *n*=4

Next, we performed in vivo animal experiments. Male mice were fed with HK and LK diets, respectively, and then injected with MLN4924 to inhibit neddylation. The results revealed that the expression of WNK4 and pWNK4 was significantly higher and the expression of NEDD8 was lower in the MLN4924 group than in the control group (Fig. 3a). We also found that p62 decreased in the HK group compared with the LK group (Fig. 3b), indicating that the HK group showed significantly stronger autophagy than the LK group. Our current findings reveal that autophagy and neddylation are involved in the KLHL3-dependent WNK4 degradation by potassium, but the specific mechanisms require further investigation.

## Conclusion

The current study was an investigation of different K^+^ concentrations affecting the autophagy and neddylation pathways of KLHL3-WNK4. Our findings suggest that potassium can regulate the activity of WNK4 and KLHL3, which associates with neddylation and autophagy.

## Supplementary Information

**Additional file 1.**

## Data Availability

The datasets generated or analyzed during this study are available from the corresponding author upon reasonable request. All data generated or analyzed during this study are included in this published article.

## Abbreviations

NCC: Na-Cl cotransporter
DCT: Distal convoluted tubule
WNK: With-no-lysine kinase
KLHL3: Kelch-like protein 3
CUL3: Cullin3
OSR1: Oxidative stress response kinase-1
SPAK: STE20/SPS1-related proline alanine–rich kinase
FHHt: Familial hyperkalemic hypertension
PHAII: Pseudohypoaldosteronism typeII
Keap1: Kelch-like ECH-associated protein1
Nrf2: Nuclear factor erythroid 2-related factor 2
