# Supplementary material for: KLHL3-dependent WNK4 degradation affected by potassium through the neddylation and autophagy pathway
Source: BMC Nephrol. 2023 Jul 22;24:217. doi: 10.1186/s12882-023-03257-4 (PMC10362690; doi:10.1186/s12882-023-03257-4)
Supplement: Supplementary file 1 — Additional file 1. [file 12882_2023_3257_MOESM1_ESM.docx]

Supplementary materials

Fig1a

1. WNK-myc


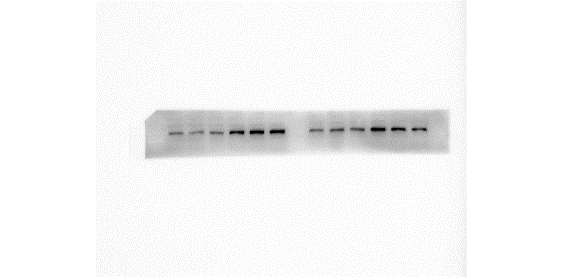

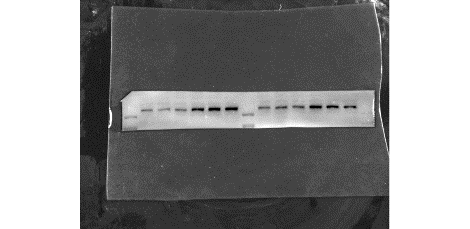


1. PWNK4


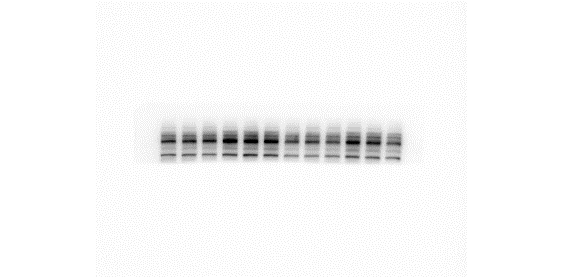

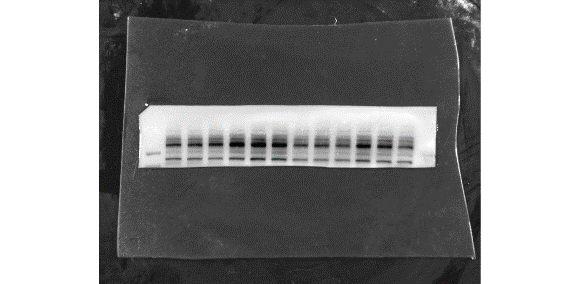


1. NEDD8-Cullin3


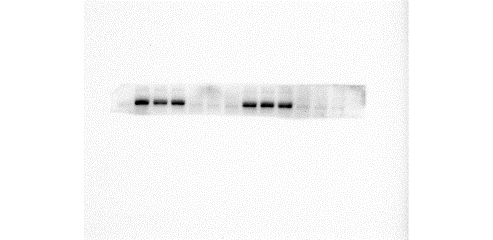

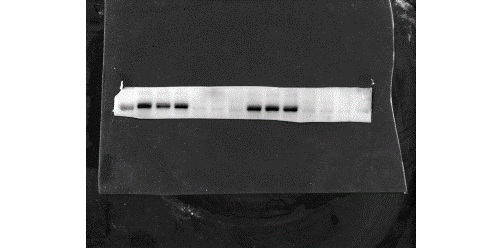


1. KLHL3-myc


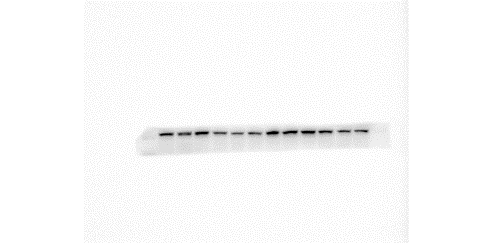

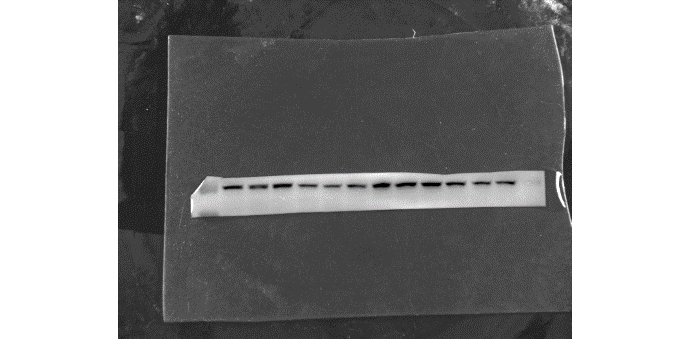


1. ACTIN


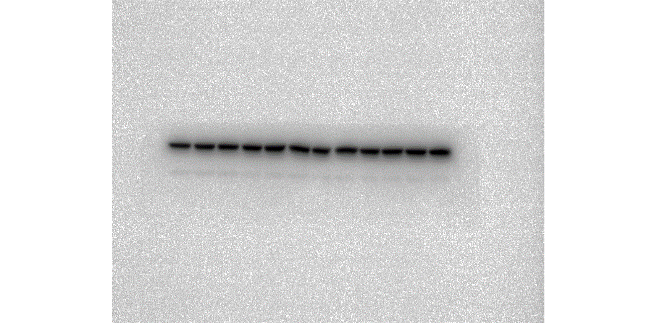

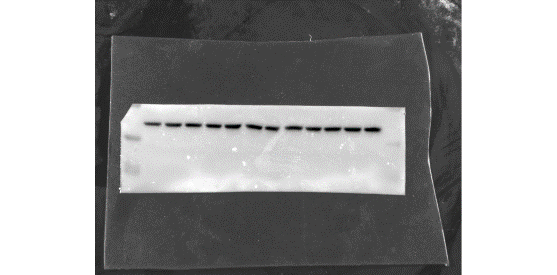


Fig1b

Attention: The 4 samples on the right side of the picture are irrelevant to this experiment, so we cut them.

1.WNK-myc


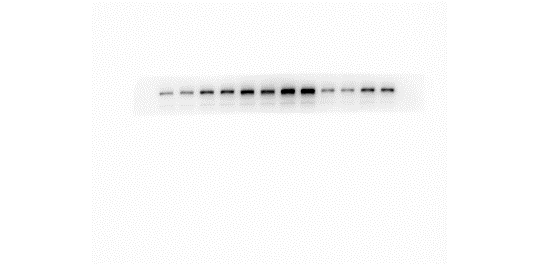

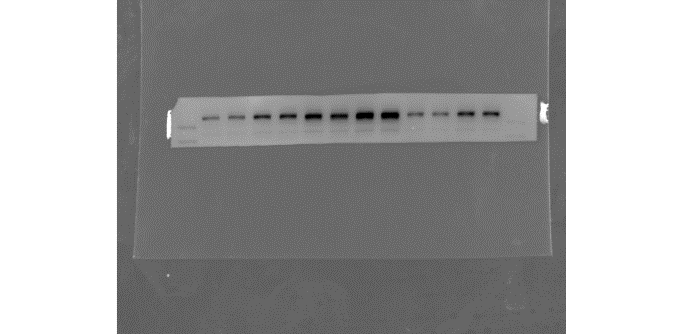


1. PWNK4


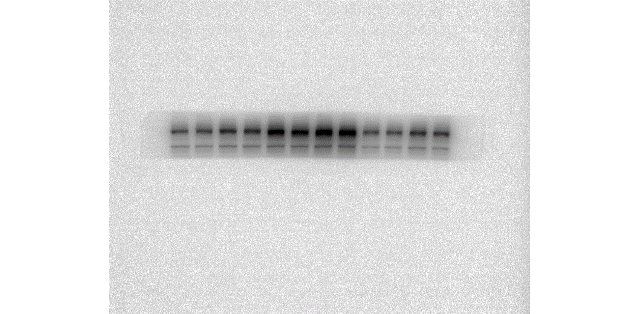

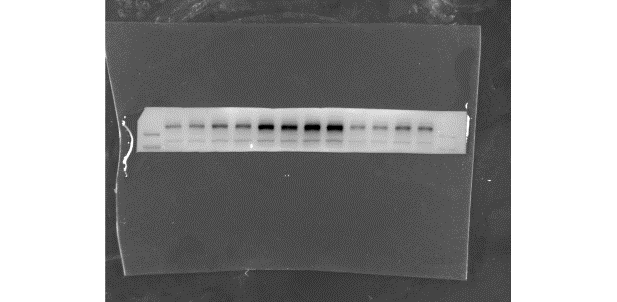


1. NEDD8-Cullin3


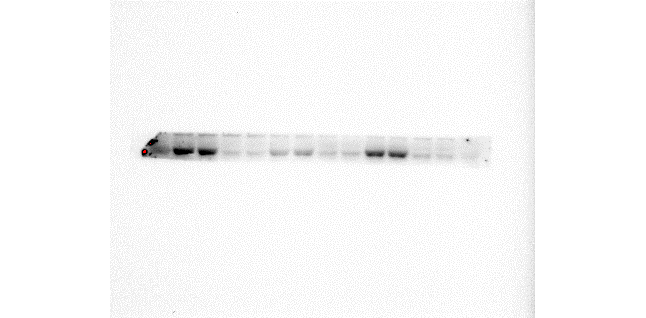

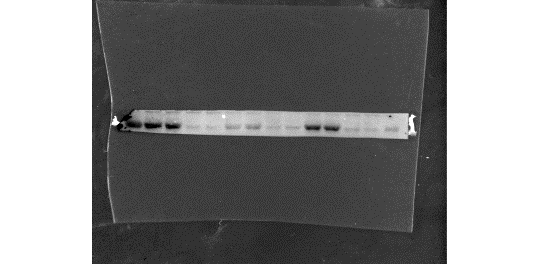


1. KLHL3-myc


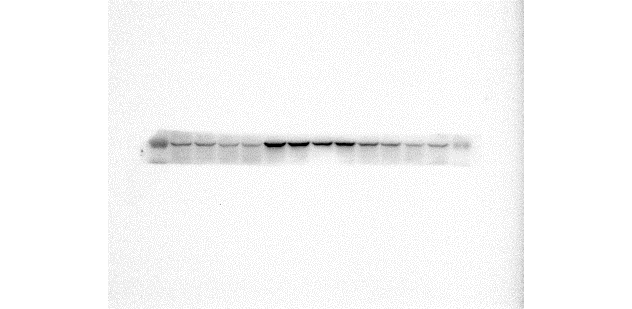

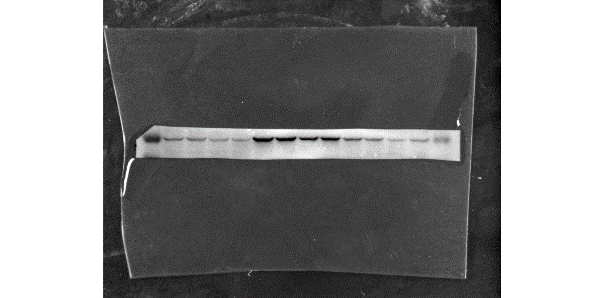


5.GAPDH


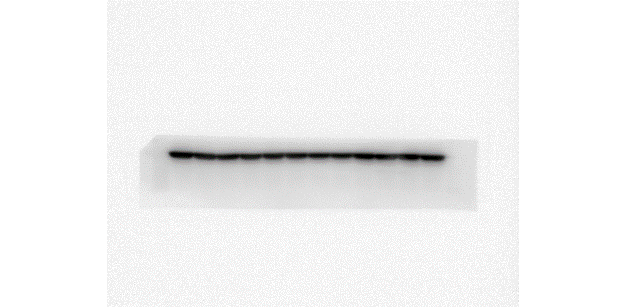

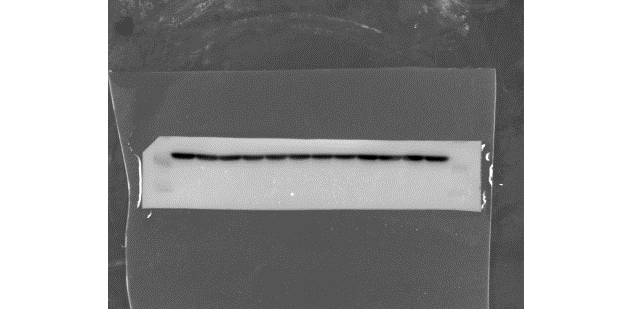


6.LC3B II


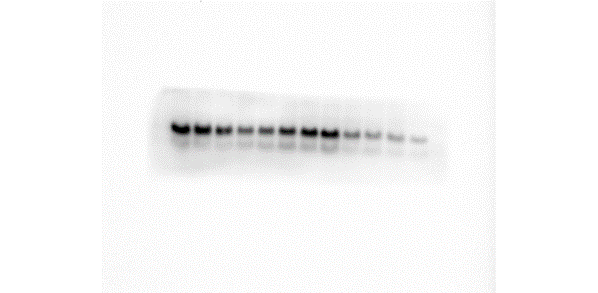

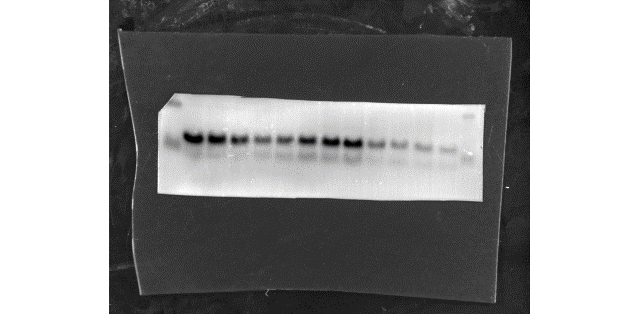


Fig1c:

Attention: The 4 samples on the right side of the picture are irrelevant to this experiment, so we cut them.

1. PWNK4


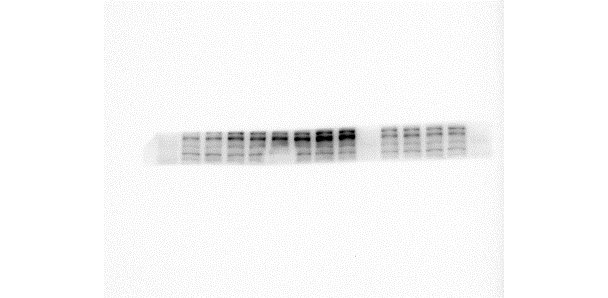

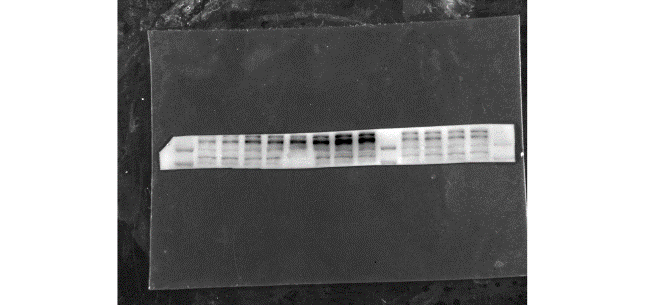


2.NEDD8-Cullin3


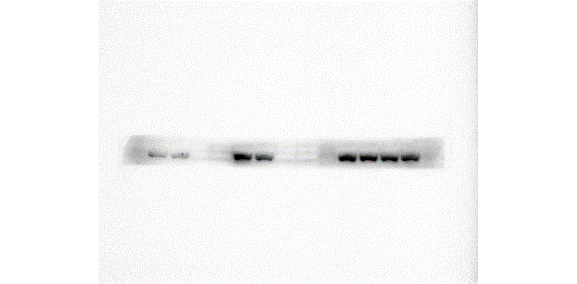

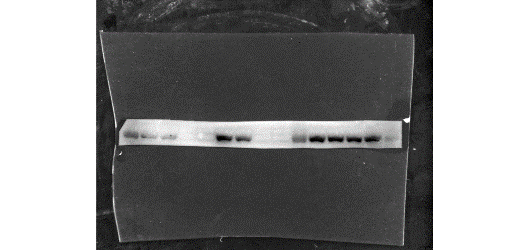


3.KLHL3-myc


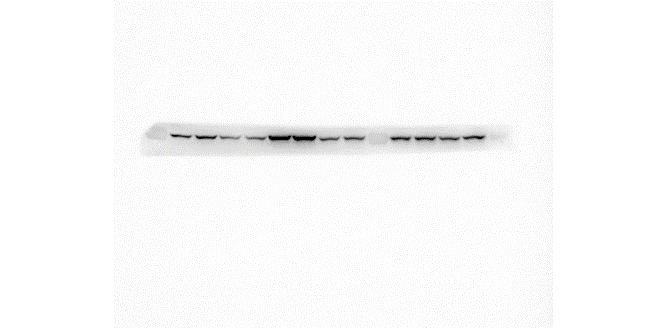

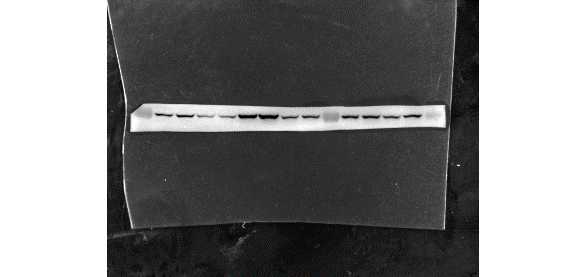


4.LC3B II


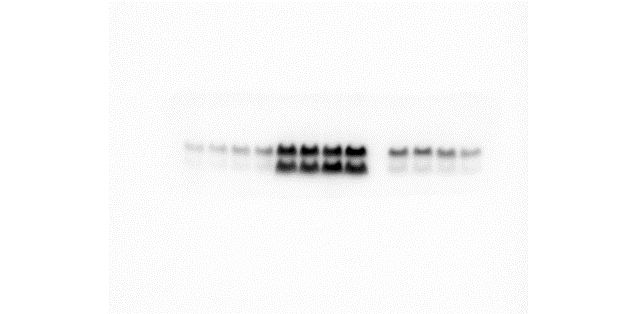

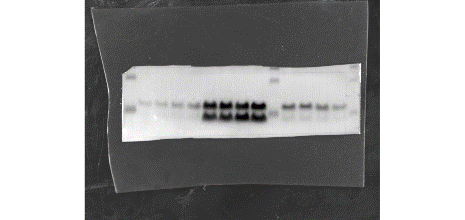


5.ACTIN


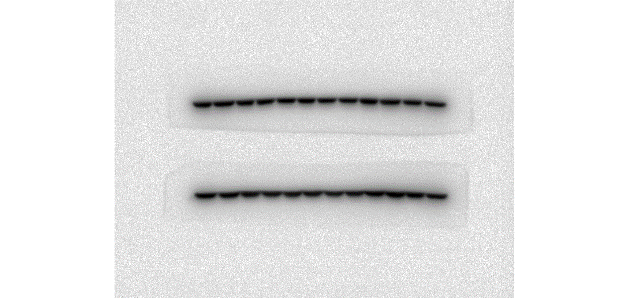

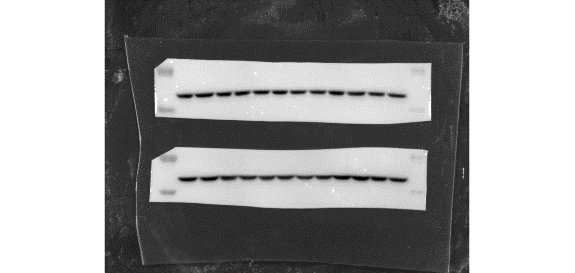


Fig2a

1. pWNK4







1. WNK4







3.NEDD8-Cullin3







1. KLHL3







1. Cullin3







1. GAPDH







Fig2b

1. LC3B II







1. Keap1







1. Nrf2







1. P62







1. Casepase3







Fig3a

Attention: the first 4 protein samples are irrelevant to this experiment, so we cut them.

1. PWNK4



**

**

1. WNK4







3.KLHL3







4.NEDD8-Cullin3







1. GAPDH


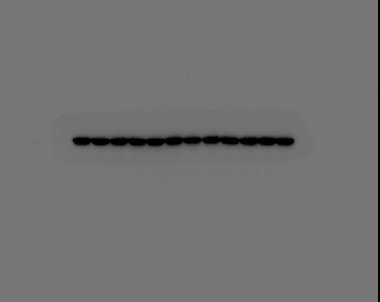






1. Cullin3







Fig3b:

Attention: the first 4 protein samples are irrelevant to this experiment, so we cut them.

1. LC3B II







1. Nrf2


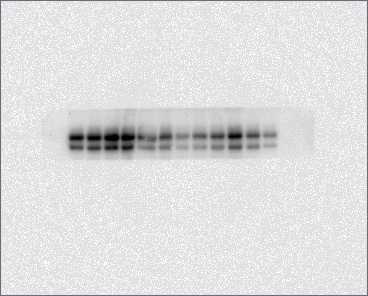






1. P62


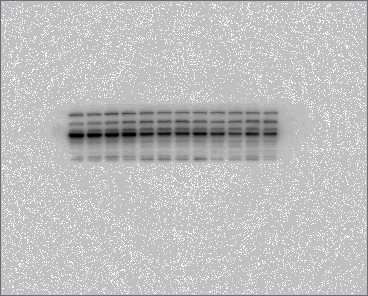






1. Keap1


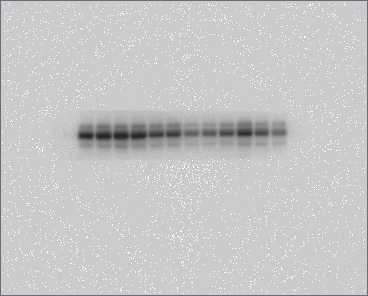






1. BECN1


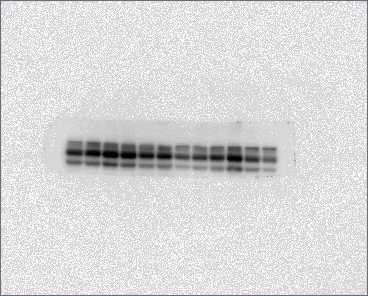






1. casepase3
